# COVID-19 Vaccination Coverage Among Adolescents Aged 12–17 Years — United States, December 14, 2020–July 31, 2021

**DOI:** 10.15585/mmwr.mm7035e1

**Published:** 2021-09-03

**Authors:** Bhavini Patel Murthy, Elizabeth Zell, Ryan Saelee, Neil Murthy, Lu Meng, Seth Meador, Kirsten Reed, Lauren Shaw, Lynn Gibbs-Scharf, A.D. McNaghten, Anita Patel, Shannon Stokley, Stephen Flores, Jonathan S. Yoder, Carla L. Black, LaTreace Q. Harris

**Affiliations:** ^1^Immunization Services Division, National Center for Immunization and Respiratory Diseases, CDC; ^2^CDC COVID-19 Response Team; ^3^Stat-Epi Associates, Inc., Ponte Vedra Beach, Florida.

Although severe COVID-19 illness and hospitalization are more common among adults, these outcomes can occur in adolescents (*1*). Nearly one third of adolescents aged 12–17 years hospitalized with COVID-19 during March 2020–April 2021 required intensive care, and 5% of those hospitalized required endotracheal intubation and mechanical ventilation (*2*). On December 11, 2020, the Food and Drug Administration (FDA) issued Emergency Use Authorization (EUA) of the Pfizer-BioNTech COVID-19 vaccine for adolescents aged 16–17 years; on May 10, 2021, the EUA was expanded to include adolescents aged 12–15 years; and on August 23, 2021, FDA granted approval of the vaccine for persons aged ≥16 years. To assess progress in adolescent COVID-19 vaccination in the United States, CDC assessed coverage with ≥1 dose* and completion of the 2-dose vaccination series^†^ among adolescents aged 12–17 years using vaccine administration data for 49 U.S. states (all except Idaho) and the District of Columbia (DC) during December 14, 2020–July 31, 2021. As of July 31, 2021, COVID-19 vaccination coverage among U.S. adolescents aged 12–17 years was 42.4% for ≥1 dose and 31.9% for series completion. Vaccination coverage with ≥1 dose varied by state (range = 20.2% [Mississippi] to 70.1% [Vermont]) and for series completion (range = 10.7% [Mississippi] to 60.3% [Vermont]). By age group, 36.0%, 40.9%, and 50.6% of adolescents aged 12–13, 14–15, and 16–17 years, respectively, received ≥1 dose; 25.4%, 30.5%, and 40.3%, respectively, completed the vaccine series. Improving vaccination coverage and implementing COVID-19 prevention strategies are crucial to reduce COVID-19–associated morbidity and mortality among adolescents and to facilitate safer reopening of schools for in-person learning.

Data on COVID-19 vaccine administration in the United States are reported to CDC by jurisdictions, pharmacies, and federal entities through immunization information systems (IISs),^§^ the Vaccine Administration Management System (VAMS),^¶^ or direct data submission.** Adolescents aged 12–17 years with valid residence in one of 49 states or DC who received ≥1 dose of a COVID-19 vaccine during December 14, 2020–July 31, 2021, and whose data were reported to CDC by August 11, 2021, were included in this analysis.^††^ COVID-19 vaccine doses administered to persons residing in Idaho were excluded because the state has data-sharing restrictions on information reported to CDC.

Receipt of ≥1 COVID-19 vaccine dose and series completion among adolescents aged 12–17 years was calculated overall and stratified by age (12–13, 14–15, and 16–17 years), sex, and jurisdiction (49 states and DC). As of August 17, 2021, only the Pfizer-BioNTech vaccine had been authorized for use among adolescents aged 12–17 years in the United States. Moderna and Janssen (Johnson & Johnson) COVID-19 vaccines were not authorized under emergency use for this age group during the analysis period; however, for reasons that are not known, many adolescents were reported to have received these vaccines, and doses administered to adolescents were included in this analysis. Vaccination coverage by race and ethnicity was not calculated because of high rates of missing data. Population size by age group and sex was obtained from the U.S. Census Bureau’s 2019 Population Estimates Program (*3*). Second dose completion was calculated among adolescents who received ≥1 dose of a 2-dose COVID-19 vaccination series and for whom sufficient time to receive a second dose during the analysis period had elapsed.^§§^ Among adolescents who received the first dose of a 2-dose COVID-19 vaccination series, the proportions of adolescents who had already received the second dose, of those who had not received the second dose but were still within the recommended time interval to receive the second dose, and of those who had not received and were overdue for the second dose were calculated. Tests for statistical significance were not conducted because these data are reflective of the U.S. population (excluding Idaho) and were not based on population samples. All analyses were conducted using SAS software (version 9.4; SAS Institute). This activity was reviewed by CDC and was conducted consistent with applicable federal law and CDC policy.^¶¶^

As of July 31, 2021, 42.4% of adolescents aged 12–17 years had received ≥1 dose of a COVID-19 vaccine (Table 1), and 31.9% had completed the vaccination series (Table 2). Adolescent COVID-19 vaccination coverage with ≥1 dose varied by state (range = 20.2% [Mississippi] to 70.1% [Vermont]), as it did for series completion (range = 10.7% [Mississippi] to 60.3% [Vermont]), with higher vaccination coverage in the Northeast and on the West Coast and lower vaccination coverage in the South (Figure). Coverage was higher among adolescents aged 16–17 years (50.6% for ≥1 dose; 40.3% for series completion) than among those aged 12–13 years (36.0% for ≥1 dose; 25.4% for series completion) and 14–15 years (40.9% for ≥1 dose; 30.5% for series completion). Vaccination coverage was similar among males and females across all age groups.

**TABLE 1:** Receipt of ≥1 COVID-19 vaccine dose by adolescents aged 12–17 years,* by age group and sex^†^ — United States,^§^ December 14, 2020–July 31, 2021

Jurisdiction	Age group and sex, no. (%)											
	12–17 yrs			12–13 yrs			14–15 yrs			16–17 yrs		
	Total	Female	Male	Total	Female	Male	Total	Female	Male	Total	Female	Male
**United States**	**10,677,934 (42.4)**	**5,425,265 (44.1)**	**5,216,450 (40.5)**	**3,094,245 (36.0)**	**1,543,152 (36.8)**	**1,541,710 (35.0)**	**3,454,771 (40.9)**	**1,750,329 (42.2)**	**1,693,216 (39.5)**	**4,128,918 (50.6)**	**2,131,784 (53.9)**	**1,981,524 (47.1)**
Alabama	77,773 (20.6)	40,050 (22.4)	37,692 (19.0)	127,065 (17.5)	11,189 (18.7)	11,094 (16.5)	25,257 (19.6)	12,996 (20.3)	12,256 (18.9)	30,221 (24.8)	15,865 (28.7)	14,342 (21.6)
Alaska	23,706 (46.4)	11,621 (50.6)	11,788 (41.9)	14,859 (46.0)	3,279 (38.2)	3,480 (55.5)	7,627 (37.0)	3,755 (42.5)	3,789 (32.1)	9,241 (59.2)	4,587 (82.9)	4,519 (44.8)
Arizona	224,638 (38.9)	114,136 (40.9)	109,744 (36.8)	201,971 (32.3)	32,501 (32.7)	32,543 (31.7)	72,338 (37.3)	36,750 (38.9)	35,297 (35.5)	87,023 (48.1)	44,885 (52.7)	41,904 (43.7)
Arkansas	73,861 (30.3)	37,256 (31.6)	35,813 (28.6)	80,882 (25.0)	9,905 (24.7)	10,097 (24.8)	24,873 (31.2)	12,407 (30.4)	12,209 (31.5)	28,754 (34.7)	14,944 (40.3)	13,507 (29.5)
California	1,642,427 (53.2)	836,970 (55.5)	801,906 (50.8)	1,054,889 (44.3)	233,673 (45.3)	232,862 (43.2)	541,389 (52.3)	275,356 (54.7)	264,914 (49.9)	633,560 (63.6)	327,941 (67.2)	304,130 (59.8)
Colorado	222,780 (50.3)	113,015 (53.3)	109,520 (47.6)	147,908 (45.0)	33,118 (48.3)	33,343 (42.0)	73,879 (48.7)	37,316 (51.5)	36,481 (46.1)	82,383 (57.6)	42,581 (59.8)	39,696 (55.3)
Connecticut	166,941 (62.3)	84,333 (64.6)	82,242 (59.9)	87,364 (55.1)	23,935 (55.1)	24,047 (54.7)	53,242 (58.7)	27,015 (62.4)	26,116 (55.1)	65,592 (72.9)	33,383 (76.0)	32,079 (69.7)
Delaware	32,169 (45.2)	16,559 (49.0)	15,560 (41.6)	21,190 (44.0)	4,614 (50.5)	4,698 (39.0)	10,526 (37.8)	5,428 (34.9)	5,080 (41.2)	12,319 (55.7)	6,517 (71.3)	5,782 (44.6)
District of Columbia	17,256 (52.3)	8,872 (53.3)	8,325 (50.9)	11,514 (49.8)	2,965 (56.6)	2,741 (43.7)	5,356 (46.0)	2,700 (38.3)	2,637 (57.5)	6,168 (62.6)	3,207 (73.4)	2,947 (53.7)
Florida	558,957 (37.6)	286,050 (39.4)	272,548 (35.9)	514,351 (31.0)	80,517 (32.9)	78,894 (29.3)	183,765 (37.2)	93,750 (37.6)	89,911 (36.8)	215,683 (45.3)	111,783 (48.4)	103,743 (42.3)
Georgia	271,600 (30.7)	138,608 (32.6)	132,222 (28.8)	307,972 (25.5)	39,194 (26.1)	39,086 (24.8)	87,107 (29.1)	44,308 (30.9)	42,597 (27.3)	105,965 (38.3)	55,106 (41.8)	50,539 (34.9)
Hawaii	60,457 (63.7)	30,251 (67.7)	30,035 (59.8)	33,044 (52.3)	8,501 (54.1)	8,725 (50.4)	19,774 (64.0)	9,869 (74.0)	9,857 (56.0)	23,409 (75.7)	11,881 (76.0)	11,453 (74.8)
Illinois	527,953 (53.2)	268,107 (54.1)	257,707 (52.0)	331,413 (45.4)	75,084 (44.8)	74,889 (45.7)	175,184 (52.1)	88,684 (52.1)	85,790 (51.7)	202,272 (62.4)	104,339 (65.9)	97,028 (58.5)
Indiana	164,717 (29.8)	84,039 (31.5)	79,638 (28.0)	194,055 (24.6)	23,834 (25.1)	23,786 (24.0)	52,778 (29.8)	26,775 (31.6)	25,704 (27.9)	64,144 (35.4)	33,430 (38.2)	30,148 (32.2)
Iowa	88,317 (36.7)	45,436 (38.6)	42,643 (34.8)	83,053 (31.8)	13,341 (32.8)	13,058 (30.8)	28,451 (36.9)	14,440 (36.2)	13,970 (37.5)	33,421 (41.6)	17,655 (47.3)	15,615 (36.4)
Kansas	88,601 (36.4)	45,509 (38.0)	42,995 (34.9)	84,150 (31.6)	13,211 (30.7)	13,352 (32.5)	27,907 (34.8)	14,328 (40.8)	13,557 (30.1)	34,100 (43.3)	17,970 (43.1)	16,086 (43.3)
Kentucky	115,204 (32.7)	59,363 (34.5)	55,723 (31.0)	122,071 (27.8)	17,009 (27.7)	16,927 (27.9)	37,571 (33.0)	19,163 (35.5)	18,375 (30.6)	43,680 (37.6)	23,191 (40.7)	20,421 (34.4)
Louisiana	81,272 (21.9)	41,478 (23.4)	39,560 (20.3)	131,531 (17.7)	11,736 (19.3)	11,536 (16.3)	26,369 (21.6)	13,273 (22.2)	13,019 (20.9)	31,616 (26.7)	16,469 (29.1)	15,005 (24.4)
Maine	48,729 (55.1)	24,474 (59.0)	23,874 (50.9)	27,699 (53.3)	7,247 (64.3)	7,370 (44.9)	16,031 (52.4)	8,004 (54.5)	7,858 (49.4)	17,937 (59.6)	9,223 (59.6)	8,646 (59.2)
Maryland	263,433 (56.3)	132,880 (57.8)	130,206 (54.7)	163,386 (49.1)	39,948 (51.2)	40,174 (47.1)	84,806 (53.6)	42,484 (52.7)	42,206 (54.4)	98,420 (67.4)	50,448 (70.9)	47,826 (63.8)
Massachusetts	319,741 (65.7)	161,726 (68.5)	157,494 (62.9)	158,110 (59.9)	47,185 (62.7)	47,336 (57.1)	105,067 (65.2)	53,176 (64.4)	51,711 (65.7)	120,042 (71.7)	61,365 (78.3)	58,447 (65.7)
Michigan	273,071 (36.0)	139,194 (38.1)	133,776 (34.1)	254,314 (30.6)	39,045 (31.8)	38,746 (29.5)	86,078 (34.7)	43,910 (36.8)	42,132 (32.7)	109,164 (42.7)	56,239 (45.5)	52,898 (40.0)
Minnesota	198,287 (44.3)	101,571 (45.8)	95,698 (42.4)	149,301 (40.8)	30,696 (39.3)	30,025 (42.1)	60,068 (38.7)	30,610 (40.9)	29,257 (36.4)	77,289 (54.1)	40,265 (58.4)	36,416 (49.3)
Mississippi	49,940 (20.2)	25,444 (21.0)	24,454 (19.3)	86,695 (16.7)	7,162 (17.7)	7,272 (15.7)	16,559 (21.4)	8,298 (20.3)	8,245 (22.7)	18,931 (22.6)	9,984 (25.0)	8,937 (20.4)
Missouri	152,486 (32.4)	77,515 (33.0)	74,807 (31.7)	158,781 (28.9)	22,844 (29.3)	23,025 (28.5)	49,384 (31.0)	25,006 (31.7)	24,325 (30.2)	57,185 (37.5)	29,665 (38.0)	27,457 (37.0)
Montana	23,962 (30.3)	12,105 (31.5)	11,683 (28.8)	25,348 (28.9)	3,579 (28.1)	3,669 (29.1)	7,428 (28.9)	3,730 (28.5)	3,638 (28.9)	9,209 (32.9)	4,796 (38.0)	4,376 (28.5)
Nebraska	62,131 (39.2)	31,723 (39.8)	30,292 (38.5)	56,881 (33.1)	9,447 (31.5)	9,343 (34.7)	19,599 (37.1)	9,928 (40.0)	9,649 (34.5)	23,719 (48.7)	12,348 (49.6)	11,300 (47.5)
Nevada	89,835 (37.2)	46,021 (37.7)	43,775 (36.6)	85,434 (29.8)	12,717 (30.8)	12,723 (28.8)	29,148 (36.3)	14,933 (34.9)	14,202 (37.8)	35,241 (46.5)	18,371 (48.4)	16,850 (44.6)
New Hampshire	48,188 (49.5)	24,264 (49.9)	23,250 (47.7)	34,943 (38.1)	6,575 (38.3)	6,609 (37.2)	15,129 (51.1)	7,567 (47.1)	7,332 (54.2)	19,747 (60.1)	10,122 (65.7)	9,309 (53.3)
New Jersey	357,267 (52.5)	180,504 (54.7)	175,521 (50.0)	232,003 (43.8)	50,304 (42.3)	51,137 (45.2)	113,832 (50.9)	57,509 (54.3)	55,960 (47.5)	141,702 (62.9)	72,691 (69.2)	68,424 (56.9)
New Mexico	92,891 (55.1)	46,824 (55.5)	44,864 (53.1)	57,115 (49.5)	13,992 (49.8)	13,800 (47.6)	29,505 (50.9)	14,745 (49.8)	14,367 (50.7)	35,137 (65.5)	18,087 (68.0)	16,697 (61.6)
New York	651,562 (46.6)	328,743 (48.5)	319,985 (44.5)	471,237 (39.3)	91,375 (39.4)	93,069 (38.9)	205,664 (44.3)	103,529 (46.5)	101,489 (42.0)	260,904 (56.6)	133,839 (60.1)	125,427 (52.6)
North Carolina	288,722 (35.4)	147,723 (35.7)	139,514 (34.8)	280,592 (29.4)	41,310 (28.5)	40,939 (30.1)	95,543 (35.3)	48,666 (35.0)	46,473 (35.3)	110,577 (42.0)	57,747 (44.3)	52,102 (39.1)
North Dakota	13,910 (26.3)	7,084 (25.4)	6,613 (26.5)	18,993 (20.2)	1,888 (20.4)	1,894 (19.5)	4,533 (27.4)	2,290 (24.1)	2,175 (30.8)	5,535 (32.0)	2,906 (31.8)	2,544 (31.3)
Ohio	284,374 (31.9)	145,410 (33.8)	138,167 (29.9)	300,214 (27.1)	40,975 (28.2)	40,302 (26.0)	89,895 (29.9)	45,960 (31.6)	43,719 (28.2)	113,035 (38.8)	58,475 (42.1)	54,146 (35.5)
Oklahoma	92,409 (29.1)	47,313 (31.4)	44,973 (27.1)	113,915 (23.4)	13,242 (25.7)	13,346 (21.4)	29,283 (29.1)	15,020 (30.0)	14,228 (28.2)	36,505 (35.5)	19,051 (38.7)	17,399 (32.5)
Oregon	147,476 (49.3)	74,896 (49.7)	72,231 (48.8)	100,819 (43.5)	21,971 (44.0)	21,828 (42.9)	48,739 (48.1)	24,714 (47.6)	23,927 (48.3)	54,859 (56.8)	28,211 (57.6)	26,476 (55.5)
Pennsylvania	437,303 (47.7)	219,211 (48.7)	209,686 (44.8)	308,332 (41.3)	62,448 (41.8)	62,623 (39.4)	140,842 (45.2)	70,283 (46.6)	67,547 (42.1)	168,972 (56.7)	86,480 (57.7)	79,516 (53.7)
Rhode Island	42,660 (55.4)	21,683 (60.8)	20,919 (50.6)	25,863 (48.2)	6,167 (45.7)	6,290 (50.9)	13,645 (51.2)	7,024 (61.9)	6,595 (43.1)	16,544 (67.4)	8,492 (78.4)	8,034 (58.6)
South Carolina	100,830 (25.8)	51,820 (26.7)	48,946 (24.9)	135,830 (19.9)	13,591 (20.5)	13,384 (19.2)	33,001 (24.6)	17,005 (25.3)	15,977 (23.9)	40,842 (33.7)	21,224 (34.9)	19,585 (32.5)
South Dakota	24,848 (34.4)	12,468 (34.6)	11,989 (33.1)	24,483 (30.1)	3,612 (32.6)	3,661 (27.3)	8,051 (30.9)	4,073 (29.1)	3,850 (31.9)	9,439 (43.5)	4,783 (43.5)	4,478 (41.8)
Tennessee	126,159 (24.3)	65,267 (26.1)	60,591 (22.6)	185,246 (19.6)	18,164 (20.1)	18,156 (19.2)	40,295 (24.0)	20,848 (24.2)	19,407 (23.8)	49,495 (29.9)	26,255 (35.8)	23,028 (24.9)
Texas	1,028,789 (40.6)	521,461 (42.2)	506,643 (39.0)	854,580 (34.6)	147,957 (35.6)	147,505 (33.6)	330,444 (38.5)	167,302 (39.9)	162,971 (37.1)	402,745 (49.1)	206,202 (51.6)	196,167 (46.6)
Utah	129,559 (41.9)	65,495 (43.8)	63,818 (40.0)	106,783 (34.6)	18,393 (34.5)	18,549 (34.7)	39,977 (38.8)	20,029 (41.4)	19,925 (36.5)	52,615 (53.1)	27,073 (56.6)	25,344 (49.3)
Vermont	28,904 (70.1)	14,332 (74.4)	14,474 (65.9)	11,732 (75.0)	4,306 (83.5)	4,464 (67.9)	9,454 (70.3)	4,790 (91.7)	4,627 (56.3)	10,649 (66.3)	5,236 (58.9)	5,383 (75.1)
Virginia	342,958 (53.7)	173,904 (56.1)	168,793 (51.3)	222,929 (45.3)	50,509 (46.5)	50,461 (44.1)	113,259 (53.0)	57,040 (54.8)	56,153 (51.2)	128,655 (63.6)	66,355 (68.2)	62,179 (59.2)
Washington	296,782 (53.1)	149,501 (53.6)	145,592 (52.0)	192,800 (48.7)	46,631 (49.0)	46,691 (47.8)	95,740 (50.0)	47,895 (48.3)	47,255 (51.2)	107,187 (61.3)	54,975 (64.9)	51,646 (57.3)
West Virginia	38,159 (30.2)	19,127 (31.5)	18,459 (28.2)	44,298 (23.8)	5,126 (24.7)	5,263 (22.3)	12,061 (30.2)	6,090 (32.4)	5,782 (27.3)	15,564 (37.1)	7,911 (37.2)	7,414 (35.8)
Wisconsin	174,211 (39.9)	88,947 (41.5)	84,996 (38.2)	142,836 (35.8)	25,720 (38.3)	25,427 (33.6)	55,260 (36.8)	28,010 (37.0)	27,178 (36.4)	67,750 (47.1)	35,217 (49.2)	32,391 (44.9)
Wyoming	9,729 (20.4)	4,982 (21.7)	4,706 (19.0)	18,337 (15.8)	1,420 (16.5)	1,471 (15.2)	3,058 (20.9)	1,548 (19.9)	1,497 (21.8)	3,772 (25.7)	2,014 (30.8)	1,738 (21.4)

* Receipt of ≥1 COVID-19 vaccine dose is defined either as receiving at least one of the 2 doses of the Pfizer-BioNTech or Moderna vaccines or a single dose of the Janssen (Johnson & Johnson) vaccine. As of August 17, 2021, only the Pfizer-BioNTech vaccine had been authorized for use among adolescents aged 12–17 years. Moderna and Janssen COVID-19 vaccines were not authorized under emergency use for this age group during the analysis period; however, these vaccinations were included in this analysis.

^†^ Fewer than 0.5% of the records were missing information on sex.

**^§^** COVID-19 vaccine doses administered to adolescents residing in Idaho were excluded because the state has data-sharing restrictions on information reported to CDC.

**TABLE 2:** COVID-19 vaccination coverage among adolescents aged 12–17 years who completed the vaccine series,* by age group and sex^†^ — United States,^§^ December 14, 2020–July 31, 2021

Jurisdiction	Age group and sex, no. (%)											
	12–17 yrs			12–13 yrs			14–15 yrs			16–17 yrs		
	Total	Female	Male	Total	Female	Male	Total	Female	Male	Total	Female	Male
**United States**	**8,045,685 (31.9)**	**4,117,404 (33.5)**	**3,905,344 (30.3)**	**2,183,597 (25.4)**	**1,093,057 (26.0)**	**1,085,039 (24.7)**	**2,570,498 (30.5)**	**1,311,724 (31.6)**	**1,251,765 (29.2)**	**3,291,590 (40.3)**	**1,712,623 (43.3)**	**1,568,540 (37.3)**
Alabama	40,925 (10.8)	21,303 (11.9)	19,606 (9.9)	10,360 (8.2)	5,234 (8.7)	5,118 (7.6)	12,421 (9.6)	6,452 (10.1)	5,969 (9.2)	18,144 (14.9)	9,617 (17.4)	8,519 (12.8)
Alaska	18,394 (36.0)	9,066 (39.5)	9,148 (32.5)	4,947 (33.3)	2,384 (27.7)	2,522 (40.2)	5,678 (27.5)	2,778 (31.4)	2,847 (24.1)	7,769 (49.7)	3,904 (70.6)	3,779 (37.4)
Arizona	167,297 (29.0)	85,471 (30.6)	81,203 (27.3)	44,661 (22.1)	22,273 (22.4)	22,209 (21.7)	52,639 (27.1)	26,854 (28.4)	25,546 (25.7)	69,997 (38.7)	36,344 (42.6)	33,448 (34.9)
Arkansas	41,891 (17.2)	21,742 (18.4)	19,956 (15.9)	10,494 (13.0)	5,259 (13.1)	5,194 (12.7)	13,552 (17.0)	6,945 (17.0)	6,551 (16.9)	17,845 (21.5)	9,538 (25.7)	8,211 (17.9)
California	1,271,593 (41.2)	652,802 (43.3)	616,318 (39.0)	344,509 (32.7)	172,803 (33.5)	171,083 (31.7)	416,508 (40.3)	213,322 (42.4)	202,396 (38.1)	510,576 (51.2)	266,677 (54.6)	242,839 (47.7)
Colorado	185,447 (41.9)	94,420 (44.5)	90,901 (39.5)	52,056 (35.2)	25,885 (37.8)	26,150 (32.9)	61,301 (40.4)	31,074 (42.9)	30,191 (38.2)	72,090 (50.4)	37,461 (52.6)	34,560 (48.1)
Connecticut	136,730 (51.0)	69,481 (53.2)	66,983 (48.8)	36,973 (42.3)	18,513 (42.6)	18,368 (41.8)	43,625 (48.1)	22,287 (51.5)	21,253 (44.8)	56,132 (62.4)	28,681 (65.3)	27,362 (59.5)
Delaware	25,675 (36.1)	13,313 (39.4)	12,334 (33.0)	7,027 (33.2)	3,496 (38.2)	3,524 (29.3)	8,378 (30.1)	4,378 (28.2)	3,990 (32.3)	10,270 (46.4)	5,439 (59.5)	4,820 (37.2)
District of Columbia	11,239 (34.1)	5,818 (34.9)	5,393 (33.0)	3,574 (31.0)	1,847 (35.2)	1,716 (27.4)	3,607 (31.0)	1,849 (26.2)	1,748 (38.1)	4,058 (41.2)	2,122 (48.6)	1,929 (35.1)
Florida	377,443 (25.4)	194,735 (26.8)	182,570 (24.0)	98,344 (19.1)	49,892 (20.4)	48,418 (18.0)	120,847 (24.5)	62,121 (24.9)	58,694 (24.0)	158,252 (33.2)	82,722 (35.8)	75,458 (30.7)
Georgia	166,329 (18.8)	85,830 (20.2)	80,219 (17.5)	41,215 (13.4)	20,691 (13.8)	20,437 (13.0)	48,426 (16.2)	24,922 (17.4)	23,455 (15.0)	76,688 (27.7)	40,217 (30.5)	36,327 (25.1)
Hawaii	35,203 (37.1)	17,549 (39.3)	17,546 (34.9)	9,931 (30.1)	4,831 (30.7)	5,072 (29.3)	11,450 (37.0)	5,705 (42.8)	5,715 (32.5)	13,822 (44.7)	7,013 (44.9)	6,759 (44.2)
Illinois	348,478 (35.1)	179,085 (36.1)	168,328 (33.9)	95,818 (28.9)	48,301 (28.8)	47,255 (28.8)	113,863 (33.9)	58,356 (34.3)	55,143 (33.2)	138,797 (42.8)	72,428 (45.8)	65,930 (39.7)
Indiana	131,406 (23.8)	67,329 (25.2)	63,257 (22.2)	35,025 (18.0)	17,450 (18.4)	17,450 (17.6)	41,394 (23.4)	21,124 (24.9)	20,030 (21.7)	54,987 (30.4)	28,755 (32.8)	25,777 (27.6)
Iowa	70,809 (29.4)	36,654 (31.1)	34,002 (27.7)	19,670 (23.7)	9,953 (24.5)	9,692 (22.8)	22,623 (29.3)	11,540 (28.9)	11,059 (29.7)	28,516 (35.5)	15,161 (40.6)	13,251 (30.9)
Kansas	61,300 (25.2)	31,698 (26.4)	29,559 (24.0)	16,594 (19.7)	8,240 (19.2)	8,339 (20.3)	18,868 (23.5)	9,778 (27.8)	9,082 (20.2)	25,838 (32.8)	13,680 (32.8)	12,138 (32.7)
Kentucky	81,664 (23.2)	42,709 (24.8)	38,895 (21.6)	22,107 (18.1)	11,199 (18.2)	10,903 (18.0)	26,034 (22.8)	13,521 (25.1)	12,500 (20.8)	33,523 (28.8)	17,989 (31.6)	15,492 (26.1)
Louisiana	46,411 (12.5)	24,126 (13.6)	22,181 (11.4)	11,607 (8.8)	5,905 (9.7)	5,695 (8.0)	13,932 (11.4)	7,128 (11.9)	6,772 (10.9)	20,872 (17.6)	11,093 (19.6)	9,714 (15.8)
Maine	42,857 (48.5)	21,496 (51.9)	21,044 (44.8)	12,259 (44.3)	5,993 (53.1)	6,149 (37.4)	14,157 (46.2)	7,069 (48.1)	6,953 (43.7)	16,441 (54.7)	8,434 (54.5)	7,942 (54.4)
Maryland	218,233 (46.7)	110,698 (48.2)	107,376 (45.1)	62,420 (38.2)	31,169 (39.9)	31,214 (36.6)	70,372 (44.5)	35,469 (44.0)	34,851 (44.9)	85,441 (58.5)	44,060 (61.9)	41,311 (55.1)
Massachusetts	263,919 (54.2)	134,332 (56.9)	129,099 (51.5)	74,471 (47.1)	37,267 (49.5)	37,081 (44.7)	86,063 (53.4)	43,839 (53.1)	42,066 (53.5)	103,385 (61.8)	53,226 (67.9)	49,952 (56.2)
Michigan	229,551 (30.3)	117,541 (32.1)	111,939 (28.5)	61,506 (24.2)	30,932 (25.2)	30,548 (23.3)	72,163 (29.1)	36,968 (31.0)	35,175 (27.3)	95,882 (37.5)	49,641 (40.2)	46,216 (34.9)
Minnesota	174,700 (39.0)	89,821 (40.5)	84,347 (37.4)	50,776 (34.0)	25,668 (32.9)	25,006 (35.1)	56,104 (36.1)	28,844 (38.5)	27,156 (33.8)	67,820 (47.4)	35,309 (51.2)	32,185 (43.5)
Mississippi	26,576 (10.7)	13,709 (11.3)	12,846 (10.2)	6,393 (7.4)	3,182 (7.9)	3,204 (6.9)	8,134 (10.5)	4,094 (10.0)	4,033 (11.1)	12,049 (14.4)	6,433 (16.1)	5,609 (12.8)
Missouri	104,029 (22.1)	53,410 (22.7)	50,568 (21.4)	28,825 (18.2)	14,385 (18.5)	14,432 (17.8)	32,843 (20.6)	16,854 (21.4)	15,979 (19.8)	42,361 (27.8)	22,171 (28.4)	20,157 (27.1)
Montana	18,046 (22.8)	9,197 (23.9)	8,794 (21.7)	5,167 (20.4)	2,551 (20.0)	2,598 (20.6)	5,430 (21.2)	2,784 (21.3)	2,626 (20.9)	7,449 (26.6)	3,862 (30.6)	3,570 (23.2)
Nebraska	48,472 (30.6)	25,035 (31.4)	23,394 (29.8)	13,509 (23.7)	6,854 (22.9)	6,647 (24.7)	15,152 (28.7)	7,759 (31.3)	7,384 (26.4)	19,811 (40.7)	10,422 (41.9)	9,363 (39.4)
Nevada	55,558 (23.0)	28,686 (23.5)	26,854 (22.5)	14,043 (16.4)	7,011 (17.0)	7,030 (15.9)	17,412 (21.7)	8,995 (21.0)	8,411 (22.4)	24,103 (31.8)	12,680 (33.4)	11,413 (30.2)
New Hampshire	39,480 (40.5)	19,952 (41.0)	18,996 (38.9)	10,267 (29.4)	5,083 (29.6)	5,094 (28.7)	12,290 (41.5)	6,173 (38.4)	5,945 (43.9)	16,923 (51.5)	8,696 (56.4)	7,957 (45.5)
New Jersey	289,682 (42.5)	146,961 (44.6)	141,715 (40.3)	77,253 (33.3)	38,290 (32.2)	38,734 (34.3)	92,001 (41.1)	46,666 (44.1)	45,052 (38.2)	120,428 (53.4)	62,005 (59.0)	57,929 (48.2)
New Mexico	72,669 (43.1)	37,085 (44.0)	35,178 (41.7)	20,417 (35.7)	10,265 (36.5)	10,012 (34.5)	22,917 (39.6)	11,613 (39.2)	11,184 (39.5)	29,335 (54.6)	15,207 (57.2)	13,982 (51.6)
New York	537,956 (38.5)	272,326 (40.2)	263,665 (36.6)	143,966 (30.6)	71,259 (30.7)	72,385 (30.2)	169,430 (36.5)	85,566 (38.4)	83,440 (34.5)	224,560 (48.7)	115,501 (51.9)	107,840 (45.2)
North Carolina	210,162 (25.8)	108,311 (26.2)	100,839 (25.2)	55,824 (19.9)	28,001 (19.3)	27,612 (20.3)	68,736 (25.4)	35,229 (25.3)	33,228 (25.2)	85,602 (32.5)	45,081 (34.6)	39,999 (30.0)
North Dakota	10,254 (19.4)	5,257 (18.9)	4,842 (19.4)	2,516 (13.2)	1,259 (13.6)	1,219 (12.5)	3,234 (19.5)	1,628 (17.2)	1,556 (22.1)	4,504 (26.1)	2,370 (26.0)	2,067 (25.4)
Ohio	239,023 (26.8)	122,890 (28.6)	115,636 (25.0)	63,374 (21.1)	32,046 (22.0)	31,238 (20.2)	74,684 (24.8)	38,484 (26.4)	36,083 (23.3)	100,965 (34.7)	52,360 (37.7)	48,315 (31.7)
Oklahoma	61,250 (19.3)	31,546 (20.9)	29,633 (17.8)	15,691 (13.8)	7,764 (15.0)	7,913 (12.7)	18,709 (18.6)	9,633 (19.2)	9,056 (18.0)	26,850 (26.1)	14,149 (28.8)	12,664 (23.7)
Oregon	126,346 (42.3)	64,593 (42.8)	61,618 (41.6)	36,145 (35.9)	18,188 (36.4)	17,937 (35.3)	41,459 (40.9)	21,114 (40.7)	20,319 (41.0)	48,742 (50.4)	25,291 (51.7)	23,362 (49.0)
Pennsylvania	303,836 (33.1)	153,011 (34.0)	145,168 (31.0)	84,516 (27.4)	41,529 (27.8)	41,414 (26.0)	98,297 (31.6)	49,226 (32.6)	47,005 (29.3)	121,023 (40.6)	62,256 (41.5)	56,749 (38.3)
Rhode Island	35,520 (46.1)	18,100 (50.7)	17,380 (42.0)	9,733 (37.6)	4,786 (35.4)	4,938 (40.0)	11,386 (42.7)	5,862 (51.6)	5,508 (36.0)	14,401 (58.7)	7,452 (68.8)	6,934 (50.6)
South Carolina	72,130 (18.4)	37,476 (19.3)	34,621 (17.6)	17,802 (13.1)	8,967 (13.5)	8,831 (12.7)	22,947 (17.1)	11,939 (17.7)	10,996 (16.4)	31,381 (25.9)	16,570 (27.3)	14,794 (24.5)
South Dakota	16,383 (22.7)	8,318 (23.1)	7,813 (21.6)	4,264 (17.4)	2,113 (19.1)	2,108 (15.7)	5,037 (19.3)	2,585 (18.5)	2,374 (19.7)	7,082 (32.6)	3,620 (32.9)	3,331 (31.1)
Tennessee	87,019 (16.8)	45,491 (18.2)	41,307 (15.4)	22,260 (12.0)	11,200 (12.4)	11,035 (11.6)	26,342 (15.7)	13,724 (15.9)	12,597 (15.4)	38,417 (23.2)	20,567 (28.0)	17,675 (19.1)
Texas	718,918 (28.4)	369,600 (29.9)	348,945 (26.9)	193,523 (22.6)	97,354 (23.4)	96,096 (21.9)	225,520 (26.2)	115,724 (27.6)	109,695 (24.9)	299,875 (36.5)	156,522 (39.1)	143,154 (34.0)
Utah	96,759 (31.3)	49,212 (32.9)	47,466 (29.8)	25,119 (23.5)	12,578 (23.6)	12,530 (23.4)	29,095 (28.3)	14,641 (30.3)	14,449 (26.4)	42,545 (42.9)	21,993 (46.0)	20,487 (39.9)
Vermont	24,881 (60.3)	12,395 (64.3)	12,437 (56.6)	7,388 (63.0)	3,657 (70.9)	3,720 (56.6)	8,118 (60.4)	4,095 (78.4)	4,006 (48.8)	9,375 (58.4)	4,643 (52.2)	4,711 (65.7)
Virginia	283,385 (44.3)	144,360 (46.6)	138,878 (42.2)	79,268 (35.6)	39,685 (36.5)	39,546 (34.6)	93,389 (43.7)	47,282 (45.5)	46,077 (42.0)	110,728 (54.7)	57,393 (59.0)	53,255 (50.7)
Washington	245,243 (43.9)	124,122 (44.5)	119,901 (42.8)	73,427 (38.1)	36,514 (38.4)	36,573 (37.4)	79,630 (41.6)	40,075 (40.4)	39,149 (42.5)	92,186 (52.7)	47,533 (56.1)	44,179 (49.0)
West Virginia	27,203 (21.6)	13,567 (22.3)	13,174 (20.1)	6,953 (15.7)	3,372 (16.3)	3,453 (14.6)	8,505 (21.3)	4,299 (22.9)	4,066 (19.2)	11,745 (28.0)	5,896 (27.7)	5,655 (27.3)
Wisconsin	140,545 (32.2)	72,235 (33.7)	68,167 (30.6)	37,736 (26.4)	19,067 (28.4)	18,641 (24.6)	43,634 (29.0)	22,269 (29.4)	21,335 (28.6)	59,175 (41.2)	30,899 (43.1)	28,191 (39.1)
Wyoming	6,866 (14.4)	3,540 (15.4)	3,305 (13.4)	1,874 (10.2)	912 (10.6)	956 (9.8)	2,162 (14.8)	1,088 (14.0)	1,070 (15.6)	2,830 (19.3)	1,540 (23.6)	1,279 (15.7)

* Vaccine series completion was defined as receiving either both doses of the Pfizer-BioNTech or Moderna vaccines, including mismatched products between the first and second dose (i.e., Pfizer-BioNTech for the first dose and Moderna for the second dose or vice versa) or a single dose for the Janssen (Johnson & Johnson) vaccine. As of August 17, 2021, only the Pfizer-BioNTech vaccine had been authorized for use among adolescents aged 12–17 years. Moderna and Janssen COVID-19 vaccines were not authorized under emergency use for this age group during the analysis period; however, these vaccinations were included in this analysis.

^†^ Fewer than 0.5% of the records were missing information on sex.

**^§^** COVID-19 vaccine doses administered to adolescents residing in Idaho were excluded because the state has data-sharing restrictions on information reported to CDC.

**FIGURE:**
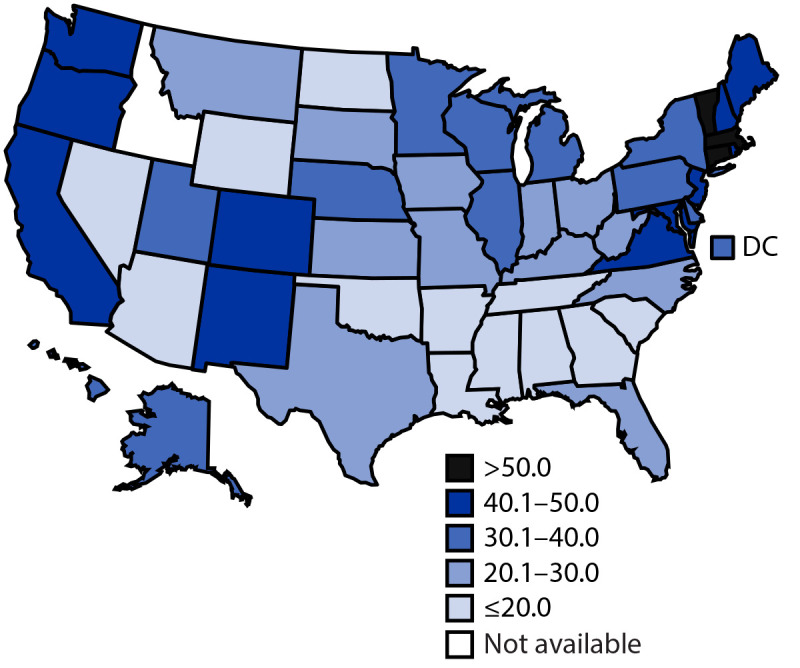
Percentage of adolescents aged 12–17 years who completed the COVID-19 vaccination series*^,†^— United States,^§^ December 14, 2020–July 31, 2021 **Abbreviation:** DC = District of Columbia. * As of August 17, 2021, only the Pfizer-BioNTech vaccine had been authorized for use among adolescents aged 12–17 years. Moderna and Janssen (Johnson & Johnson) COVID-19 vaccines were not authorized under emergency use for this age group during the analysis period; however, many adolescents had documentation of receipt of these vaccines. Thus, these vaccine doses were included in this analysis if they were administered to adolescents aged 12–17 years. ^†^ Series completion was defined as receipt of either both doses of the Pfizer-BioNTech or Moderna vaccines, including those who might have received mismatched products between the first and second dose (i.e., Pfizer-BioNTech for the first dose and Moderna for the second dose or vice versa) or a single dose of the Janssen vaccine. ^§^ COVID-19 vaccine doses administered to adolescents residing in Idaho were excluded because the state has data-sharing restrictions on information reported to CDC.

Overall, 86.8% of adolescents aged 12–17 years who received the first dose of a 2-dose COVID-19 vaccination series*** received the second dose within the recommended interval. A total of 2.4% had not received the second dose but were within the allowable interval, and 10.8% were overdue for the second dose (i.e., >42 days since receipt of the first dose) (Supplementary Table, https://stacks.cdc.gov/view/cdc/109000).

## Discussion

Among all U.S. adolescents aged 12–17 years who received the first dose of a 2-dose COVID-19 vaccine series, the vast majority received the second dose, indicating high adherence to completing the COVID-19 vaccine series. However, as of July 31, 2021, only 42.4% of adolescents had received ≥1 dose of a COVID-19 vaccine, and fewer than one third (31.9%) had completed the vaccination series. Further, vaccination coverage varied widely by state, with those in the Northeast and on the West Coast reporting the highest COVID-19 vaccination coverage among adolescents. Vaccination coverage also varied widely by age group, with reported coverage higher among those aged 16–17 years compared with those aged 12–15 years. This is likely because the older age group has been vaccine-eligible for a longer period (i.e., since December 2020).

After the start of the COVID-19 pandemic, many schools shifted to virtual or hybrid learning. Because in-person learning fosters social and emotional development,^†††^ safely returning to schools for in-person learning remains a goal. However, given the rapid emergence and spread of the highly transmissible B.1.617.2 (Delta) variant of SARS-CoV-2, the virus that causes COVID-19, and the increase in cases and hospitalizations among children and adolescents (*1*), ensuring high adolescent vaccination coverage is crucial to a safer return to the classroom. Unvaccinated or undervaccinated adolescents can become ill with COVID-19 and spread the SARS-CoV-2 virus in schools, and by extension, in local communities, placing other populations at risk. School systems can consider implementing layered prevention strategies consistent with CDC’s guidance for COVID-19 prevention in schools, including universal indoor masking regardless of vaccination status, improving ventilation, screening testing, physical distancing where feasible, and contact tracing in combination with quarantine and isolation. As the 2021–22 school year begins, concerted public health efforts are needed to increase COVID-19 vaccination coverage among adolescents in addition to implementing COVID-19 prevention strategies based on community transmission.

Public health practitioners can use various measures to increase adolescent COVID-19 vaccination coverage. Building on lessons from the public-private partnership between CDC and retail pharmacies in the Federal Retail Pharmacy Partnership^§§§^ regarding vaccination clinics offered for selected population groups at different times throughout the response (*4*), local public health agencies and pharmacies could partner with school districts and school systems to provide COVID-19 vaccinations to students at schools. Vaccine administration on site at schools is an effective, evidence-based intervention that improves childhood and adolescent vaccination rates for routinely recommended vaccines (*5*). State and local governments, school administrators, community leaders, health care professionals, and public health practitioners can facilitate safer return to schools and improve equity among sociodemographic groups by prioritizing COVID-19 vaccination among adolescents and incorporating on-site school vaccinations for eligible students (*6*,*7*). In addition, on-site vaccination clinics might also be planned in coordination with other school-based vaccination programs, such as those for seasonal influenza and routine adolescent vaccination.

Concerted outreach can help inform adolescents and their parents about the importance of COVID-19 vaccination. Effective outreach with tailored communication could help improve vaccine confidence, acceptance, and coverage among adolescents and their parents. In a recent report, only 56% of parents of unvaccinated adolescents aged 12–17 years expressed intent for their adolescent to receive a COVID-19 vaccine (*8*). Given that parental vaccination status is a marker for adolescent vaccination status,^¶¶¶^ vaccine hesitancy or antivaccination sentiments among parents might directly lead to missed opportunities to vaccinate adolescents (*9*). Among adolescents and their parents who were surveyed about their intent to receive a COVID-19 vaccine, many reported that having more information about the safety and efficacy of COVID-19 vaccines would increase their likelihood of receiving a vaccine (*8*). Public health practitioners can use multimodal outreach efforts involving a variety of traditional and social media platforms to engage adolescents and their parents to improve vaccination acceptance and coverage. Further, state and local governments can consider strategies that encourage receipt by adolescents of all vaccines recommended by the Advisory Committee on Immunization Practices, especially given the declines in routine childhood and adolescent vaccinations during the pandemic *(**10**)*.

The findings in this report are subject to at least five limitations. First, vaccination coverage rates were aggregated and analyzed only at the state level. Calculating coverage at more specific levels (e.g., by county or urban-rural classification) could potentially identify geographic areas with low vaccination coverage rates. Second, because Idaho was excluded from the analysis, the findings are not representative of the entire United States. Third, adolescents who received COVID-19 vaccines from different entities that used different methods for submitting data (e.g., if the first dose was administered at a pharmacy and the second dose was given at a mass vaccination site) might not have their first and second doses linked, which could have led to underestimation of the percentage of adolescents who completed the vaccination series. Fourth, if an adolescent had inadvertently received a different recipient ID when receiving their second dose, first and second doses could not be linked. Finally, vaccination coverage could not be calculated on the basis of race and ethnicity because of incomplete reporting.

An estimated 2 million COVID-19 cases and approximately 300 associated deaths have been reported among children aged 5–17 years since the start of the COVID-19 pandemic (*1*). As persons in younger age groups become eligible for COVID-19 vaccination, public health practitioners, health care professionals, school administrators, and state and local governments can use evidence-based practices to decrease barriers to vaccination and increase confidence in COVID-19 vaccines, which can help facilitate the safer return to in-person learning at schools and ultimately reduce COVID-19–associated morbidity and mortality.

**Summary**

**What is already known about this topic?**

Although more common among adults, severe COVID-19 illness and hospitalization occur among adolescents.

**What is added by this report?**

As of July 31, 2021, coverage with ≥1 dose of COVID-19 vaccine among adolescents aged 12–17 years was 42%, and 32% had completed the series. Series completion rates varied widely by state, ranging from 11% to 60%, and was 25% for adolescents aged 12–13 years, 30% for those aged 14–15 years, and 40% for those aged 16–17 years.

**What are the implications for public health practice?**

Improving adolescent COVID-19 vaccination coverage is crucial to reduce COVID-19–associated morbidity and mortality among adolescents and can help facilitate safer reopening of schools for in-person learning.

## Footnotes

* Receipt of ≥1 COVID-19 vaccine dose is defined as having received either ≥1 of the 2 Pfizer-BioNTech or Moderna vaccine doses, or a single dose of the Janssen (Johnson & Johnson) vaccine. As of August 17, 2021, only the Pfizer-BioNTech vaccine had been authorized for use among adolescents aged 12–17 years. Moderna and Janssen COVID-19 vaccines were not authorized under emergency use for this age group during December 14, 2020–July 31, 2021. However, doses of these vaccines administered to persons aged 12–17 years were included in this analysis. During February 27, 2021–July 31, 2021, a total of 21,919 adolescents aged 12–17 years were reported to have received 1 dose of the Janssen COVID-19 vaccine. During December 14, 2021–July 31, 2021, a total of 27,226 adolescents aged 12–17 years were reported to have received only the first dose of the Moderna COVID-19 vaccine; 66,032 adolescents aged 12–17 years were reported to have received both doses of the Moderna COVID-19 vaccine; 2,190 were reported to have received Pfizer-BioNTech for the first dose but Moderna for the second dose; and 5,726 were reported to receive Moderna for the first dose but Pfizer-BioNTech for the second dose.

† Series completion was defined as receipt of either both doses of the Pfizer-BioNTech or Moderna vaccines, including those that might have received mismatched products between the first and second dose (i.e., Pfizer-BioNTech for the first dose and Moderna for the second dose or vice versa) or a single dose of the Janssen vaccine.

§ IISs are confidential, computerized, population-based systems that collect and consolidate vaccination data from providers in 64 public health jurisdictions and can be used to track administered vaccines and measure vaccination coverage. The 64 IIS jurisdictions comprise the 50 U.S. states, eight U.S. territories and freely associated states (Puerto Rico, U.S. Virgin Islands, American Samoa, Commonwealth of the Northern Mariana Islands, Guam, Marshall Islands, Palau, and the Federated States of Micronesia), and six local jurisdictions (Chicago, IL; Houston, TX; San Antonio, TX; Philadelphia, PA; New York City, NY; and Washington, DC).

¶
https://www.cdc.gov/vaccines/covid-19/reporting/vams/program-information.html

**
https://www.cdc.gov/vaccines/covid-19/reporting/overview/IT-systems.html

†† Providers are required to document vaccination in their medical records within 24 hours of administration and submit these data to their jurisdiction’s IIS within 72 hours of administration.

§§ Although the recommended interval between doses is 21 days for the Pfizer-BioNTech vaccine, adolescents whose second doses were administered as early as 17 days after the first dose or >21 days after the first dose were considered to have completed the vaccination series. As of August 17, 2021, the Moderna COVID-19 vaccine had not been authorized for use among adolescents. However, the interval between the 2 Moderna COVID-19 vaccine doses was assessed in the analysis. Although the recommended interval between doses is 28 days for the Moderna vaccine, second doses received as early as 24 days after the first dose or >28 days after the first dose were considered to complete the vaccine series.

¶¶ 45 C.F.R. part 46.102(l)(2); 21 C.F.R. part 56; 42 U.S.C. Sect. 241(d); 5 U.S.C. Sect. 552a; 44 U.S.C. Sect. 3501 et seq.

*** Among persons who received their first dose on or before July 6, 2021, for Pfizer-BioNTech (i.e., >25 days between the first dose and July 31, 2021) or June 29, 2021, for Moderna (i.e., >32 days between the first dose and July 31, 2021). Percentages might not sum to 100% because persons who were not yet due for the second dose were excluded from this analysis.

†††
https://www.cdc.gov/coronavirus/2019-ncov/community/schools-childcare/k-12-guidance.html#anchor_1625661937509

§§§
https://www.cdc.gov/vaccines/covid-19/retail-pharmacy-program/index.html

¶¶¶
https://www.kff.org/coronavirus-covid-19/poll-finding/kff-covid-19-vaccine-monitor-parents-and-the-pandemic
